# In Vivo Assessment of Plasma Gel: Regenerative Potential and Limitations as a Filler

**DOI:** 10.1111/jocd.16765

**Published:** 2025-02-07

**Authors:** Hao Cheng, Ju Li, Yong Zhao, Xun Xia, Yan Li

**Affiliations:** ^1^ Department of Medical Aesthetics The First Affiliated Hospital of Chengdu Medical College Chengdu Sichuan Province China; ^2^ Department of Stomatology The First Affiliated Hospital of Chengdu Medical College Chengdu Sichuan Province China; ^3^ Department of Neurosurgery The First Affiliated Hospital of Chengdu Medical College Chengdu Sichuan Province China

## Abstract

**Background:**

Plasma gel, also known as plasma‐rich in growth factors (PRGF) gel or platelet‐rich gel, is a novel application of platelet‐rich plasma (PRP), created by heating platelet‐poor plasma (PPP) to induce denaturation, forming a gel‐like substance. This gel is then combined with PRP to create a new injectable biomaterial that offers both the regenerative benefits of PRP and structural support due to its gel‐like consistency. Plasma gel has been increasingly used not only for its biological regenerative properties but also as a cosmetic filler to enhance tissue volume and improve skin aesthetics. It shows promise in facial rejuvenation by improving skin texture and elasticity, and reducing the appearance of wrinkles. However, the detailed in vivo behavior of plasma gel, particularly its absorption rate and regenerative efficacy, remains underexplored.

**Aims:**

This study aims to investigate the in vivo behavior of plasma gel, focusing on its absorption rate and its ability to stimulate tissue regeneration. Specifically, we aim to assess how quickly plasma gel is absorbed after subcutaneous injection and to evaluate its effects on collagen production and neovascularization using a nude mouse model.

**Methods:**

Plasma gel was prepared by heating PPP to create a gel‐like substance, which was then mixed with PRP. This mixture was injected subcutaneously into nude mice. The absorption of the gel was monitored over time by measuring the remaining volume at different time points. Tissue samples were analyzed using Masson's trichrome staining to detect collagen and CD31 immunohistochemical staining to assess neovascularization to understand the regenerative effects induced by plasma gel.

**Results:**

The study found that plasma gel is absorbed rapidly, with approximately 50% of the volume disappearing within the first week and almost complete absorption by eight weeks. Despite this rapid absorption, significant increases in collagen deposition and new blood vessel formation were observed, indicating strong regenerative properties even after the gel had been largely absorbed.

**Conclusions:**

The main findings of this study suggest that while plasma gel is quickly absorbed and may not be suitable for long‐term volumizing effects, it shows significant potential for temporary volume enhancement and as a biostimulatory agent. Plasma gel's ability to promote collagen production and neovascularization makes it valuable in clinical applications where temporary support and long‐term tissue regeneration are desired. Future research should focus on ways to extend the retention time of plasma gel and enhance its regenerative effects, potentially through modifications to its formulation or combination with other bioactive substances.

## 1 Introduction

Plasma gel, also referred to as plasma rich in growth factors (PRGF) gel or platelet‐rich gel, represents a novel extension of platelet‐rich plasma (PRP) technology. It is produced by heating platelet‐poor plasma (PPP), which causes the proteins to denature and form a gel‐like material. This gel is then combined with PRP to develop a new type of injectable biomaterial. This material not only retains the regenerative properties associated with PRP but also offers enhanced structural support due to its gel‐like consistency, making it suitable for both tissue regeneration and cosmetic filling purposes. Plasma gel is utilized for various applications, including dermal fillers and tissue regeneration [1]. The autologous nature of plasma gel, which minimizes the risk of adverse reactions and enhances biocompatibility, makes it an attractive option for aesthetic and therapeutic purposes [2].

The development of plasma gel for cosmetic filling and tissue regeneration addresses the limitations of traditional fillers like hyaluronic acid and collagen, which often degrade quickly and may cause allergic reactions. Plasma gel, on the other hand, provides a biocompatible and patient‐specific alternative that supports tissue regeneration and offers structural support when used as a dermal filler [1, 3, 4].

Recent advancements have improved the mechanical properties and regenerative capabilities of plasma gel. For instance, incorporating composite materials such as alginate with plasma has enhanced the structural integrity and stiffness of the gels, making them more suitable for tissue engineering applications. These composite gels exhibit significantly increased stiffness and better support for cell proliferation and differentiation, essential for effective tissue regeneration [5, 6].

Plasma gel has demonstrated significant potential in promoting collagen synthesis and enhancing wound healing, making it a valuable tool in regenerative medicine. When combined with PRP, plasma gel accelerates wound healing and tissue repair by providing a scaffold that supports cellular infiltration and matrix deposition. This dual application not only enhances the structural support but also amplifies the regenerative effects of PRP. Furthermore, studies have shown that when PRGF gel is co‐cultured with fibroblasts in vitro, it significantly promotes cell proliferation and collagen synthesis, contributing to improved skin quality and accelerated healing. This ability to enhance both cellular activity and extracellular matrix formation underlines the versatility of plasma gel as a therapeutic agent in a variety of clinical applications [7, 8, 9, 10].

Our study represents the first quantification of plasma gel absorption changes in vivo, providing crucial data on subcutaneous collagen regeneration. We injected human plasma gel subcutaneously in nude mice and monitored its support and volume absorption over time, as well as its effect on subcutaneous collagen regeneration. The results revealed that plasma gel absorption was rapid, with the gel volume reducing by half within one week and almost completely absorbed by day 28. This indicates that while plasma gel's filling effect is initially effective, its duration is relatively short, necessitating future research to develop methods for prolonging its persistence.

On the other hand, the regenerative effects of plasma gel were validated, as we observed a significant increase in subcutaneous collagen in the mice. This highlights the potential of plasma gel in promoting tissue regeneration, even though its short duration as a filler remains a limitation that needs to be addressed.

In conclusion, while plasma gel shows promising regenerative properties, its rapid absorption limits its effectiveness as a long‐term filler. Future research should focus on optimizing plasma gel formulations to enhance their longevity while maintaining their regenerative benefits. This study provides essential insights for the clinical application of plasma gel, paving the way for improved materials in cosmetic and therapeutic settings.

## 2 Materials and Methods

### 2.1 Plasma Gel Preparation

Plasma gel was prepared from venous blood drawn from healthy volunteers. The following procedure outlines the steps involved in the preparation of the plasma gel:

1. Blood Collection: Venous blood was collected from volunteers into 9 mL centrifuge tubes without any anticoagulants.

2. Centrifugation: The blood samples were subjected to a multi‐step centrifugation process to separate the components:

Step 1: The centrifuge was accelerated for 30 s to reach a speed of 2700 rpm (692 g).

Step 2: The blood was centrifuged at 2700 rpm (692 g) for 2 min.

Step 3: The speed was reduced to 2400 rpm (547 g) and centrifuged for 4 min.

Step 4: The speed was increased to 2700 rpm (592 g) and centrifuged for another 4 min.

Step 5: The speed was further increased to 3000 rpm (855 g) and centrifuged for 3 min.

Step 6: The centrifuge was decelerated for 36 s until it came to a stop.

3. Layer Separation: Following centrifugation, the blood separated into three distinct layers:

Top layer: Platelet‐poor plasma (PPP).

Middle layer: Concentrated growth factors (CGF).

Bottom layer: Red blood cells (RBCs).

4. Extraction of Layers: The PPP and CGF layers were carefully extracted and collected separately.

5. Preparation of Plasma Gel:

The extracted PPP was heated at 75°C for 10 min.

The heated PPP was then allowed to cool, resulting in the formation of a gel‐like substance, referred to as plasma gel.

6. Formation of Final Injectable Plasma Gel:

The cooled plasma gel (derived from PPP) was mixed with the CGF layer in a ratio of 4:1.

The final mixture of plasma gel and CGF formed a biologically functional injectable plasma gel, ready for use in experimental procedures.

This preparation method ensures that the plasma gel retains its biological properties, making it suitable for applications in tissue regeneration and cosmetic treatments.

### 2.2 Animal and Establishment of Animal Models

The animal experiments were conducted using nude mice, with the study receiving approval from the Ethics Committee of Chengdu Medical College. The mice were divided into seven groups, with each group consisting of three mice. The groups were designated based on the timeline for tissue collection post‐injection: 1 day, 1 week, 2 weeks, 3 weeks, 4 weeks, 6 weeks, and 8 weeks.

To establish the animal model, the prepared plasma gel was loaded into a 2.5 mL syringe fitted with a 27G sharp needle. Each mouse received a subcutaneous injection of 1 mL of the plasma gel into the dorsal subcutaneous space.

At each designated time point, the mice were euthanized, and the implanted plasma gel was carefully extracted. The primary objective was to measure the weight of the extracted implants to assess the absorption rate and retention of the plasma gel.

Following the extraction of the implants, the surrounding skin and subcutaneous tissues were collected for further analysis. These tissues were subjected to histological staining to evaluate the structural and cellular changes, with a focus on the regeneration of collagen and other tissue responses around the implant site.

### 2.3 Histological Analysis

Subcutaneous tissue samples extracted from plasma gel injection sites underwent histological analysis. The harvested tissues were processed for paraffin embedding, sectioned, and subsequently stained using three distinct staining techniques to evaluate various aspects of tissue response and regeneration.

Hematoxylin and Eosin (H&E) staining was employed to examine the general histological architecture and cellular response at the injection site. This technique provided detailed information on tissue morphology, inflammatory processes, and cellular infiltration surrounding the plasma gel implants.

Masson's trichrome staining was utilized to assess collagen deposition and fibrosis. This staining method facilitated the differentiation of collagen fibers from other tissue components, enabling the evaluation of the extent and quality of the collagen regeneration induced by the plasma gel.

CD31 immunohistochemical staining was performed to detect endothelial cells and assess neovascularization at the injection site. CD31, a marker for endothelial cells, indicates the formation of new blood vessels, which is crucial for tissue regeneration and healing.

The combined application of these three staining methods allowed for a comprehensive assessment of tissue response and regeneration following plasma gel injection.

### 2.4 Statistical Analysis

Analysis of variance (ANOVA) and t tests were performed using GraphPad Prism 9.0 (GraphPad Software Inc., San Diego, CA, USA) and SPSS (IBM Inc., Armonk, NY, USA). Data are reported as the mean ± standard deviation. *p* < 0.05 indicated that a difference was statistically significant.

## 3 Results

### 3.1 Absorption Kinetics of Plasma Gel

**FIGURE 1:**
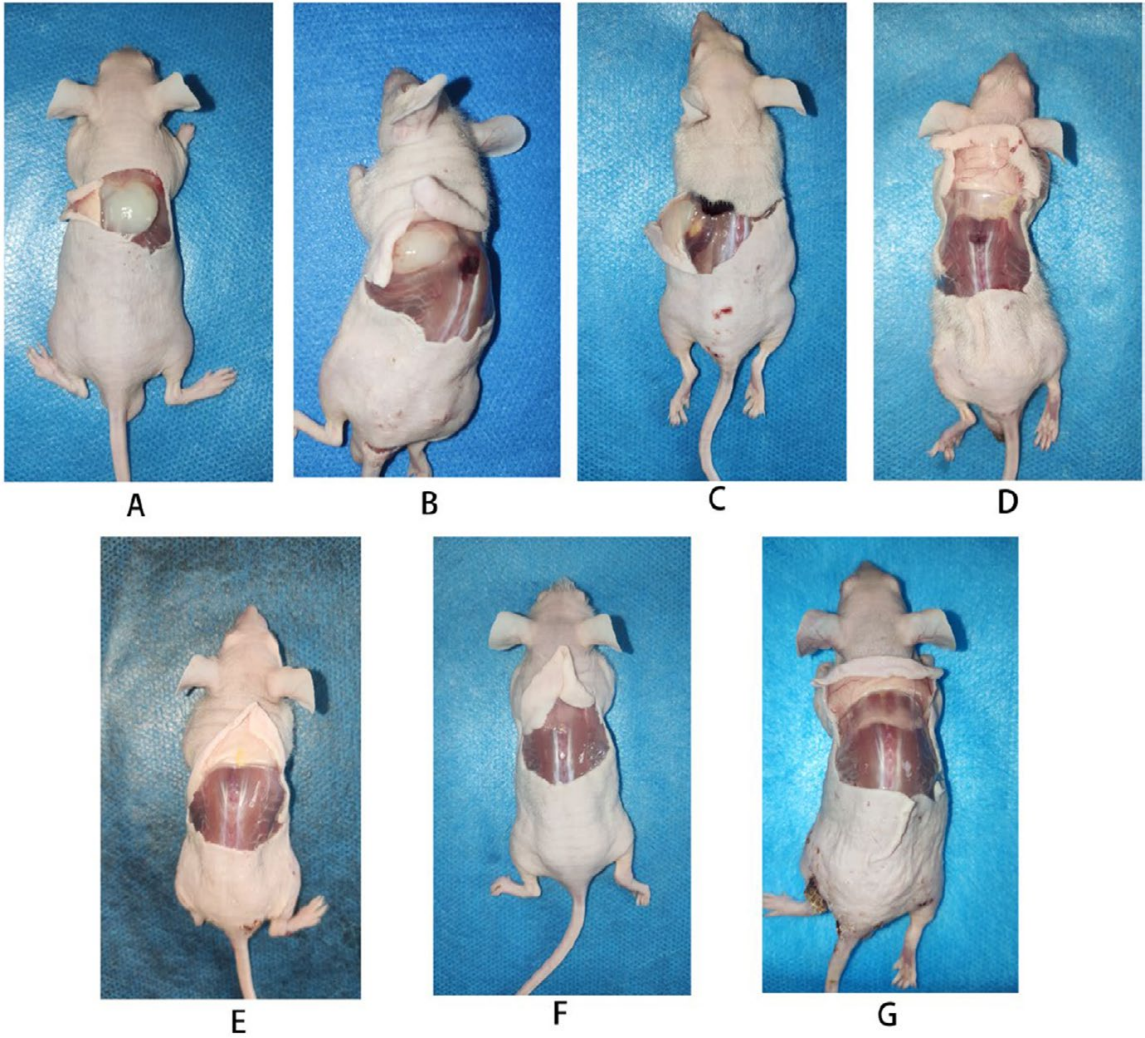
Macroscopic images of nude mouse dorsal tissue at various time points following subcutaneous injection of plasma gel. (A) 1 day post‐injection, (B) 1 week post‐injection, (C) 2 weeks post‐injection, (D) 3 weeks post‐injection, (E) 4 weeks post‐injection, (F) 6 weeks post‐injection, (G) 8 weeks post‐injection. These images depict the visible changes in the injection site over time, demonstrating the gel's persistence and the tissue response at different stages of the study.

**FIGURE 2:**
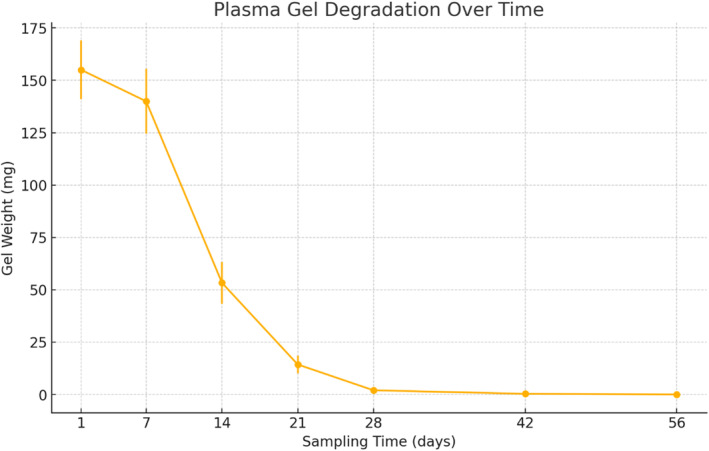
Visualization of residual gel weight in mouse subcutaneous tissue over time post‐injection. The *x*‐axis represents the time points of tissue collection after gel injection (1 day, 1 week, 2 weeks, 3 weeks, 4 weeks, 6 weeks, and 8 weeks). The *y*‐axis shows the average residual weight of the gel (mean ± standard deviation) at each time point. This graph illustrates the degradation kinetics of the gel, with a noticeable reduction in weight over time, indicating progressive degradation.

Nude mice were euthanized at various time points post‐injection of the gel, and images of the gel and surrounding tissue at the time of collection are shown in Figure 1. The absorption rate of subcutaneously injected plasma gel was quantified by measuring the weight of the remaining gel at various time points post‐injection (Figure 2). The initial average weight of the injected gel was 155.00 ± 11.43 mg (mean ± SD, *n* = 3) at day 1. A rapid decrease in gel weight was observed within the first week, with the average weight reducing to 140.00 ± 12.68 mg by day 7, representing approximately 90% of the initial volume.

The rate of absorption accelerated dramatically in the subsequent weeks. By week 2, the average gel weight had decreased to 53.30 ± 8.18 mg, approximately 34% of the initial weight. This trend continued, with the average weight further reducing to 14.30 ± 3.40 mg (9.2% of initial weight) by week 3, and only 2.00 ± 0.82 mg (1.3% of initial weight) remaining by week 4.

At weeks 6 and 8, the gel was almost completely absorbed, with weights of 0.33 ± 0.47 mg and 0.00 mg, respectively. These results demonstrate a biphasic absorption pattern, characterized by an initial gradual decrease followed by a rapid absorption phase, leading to near‐complete disappearance of the gel by 6 weeks post‐injection (Table 1).

**TABLE 1:** Residual weight of gel in mouse subcutaneous tissue at various time points post‐injection. The table shows the average residual weight (mean ± standard deviation) of the gel collected at each time point. The measurements indicate the rate of gel degradation over time.

Group	Mean weight (mg)
1 day	155.00 ± 11.43
1 week	140.00 ± 12.68
2 weeks	53.30 ± 8.18
3 weeks	14.30 ± 3.40
4 weeks	2.00 ± 0.82
6 weeks	0.33 ± 0.47
8 weeks	0.00

### 3.2 Collagen Regeneration in Subcutaneous Tissue

**FIGURE 3:**
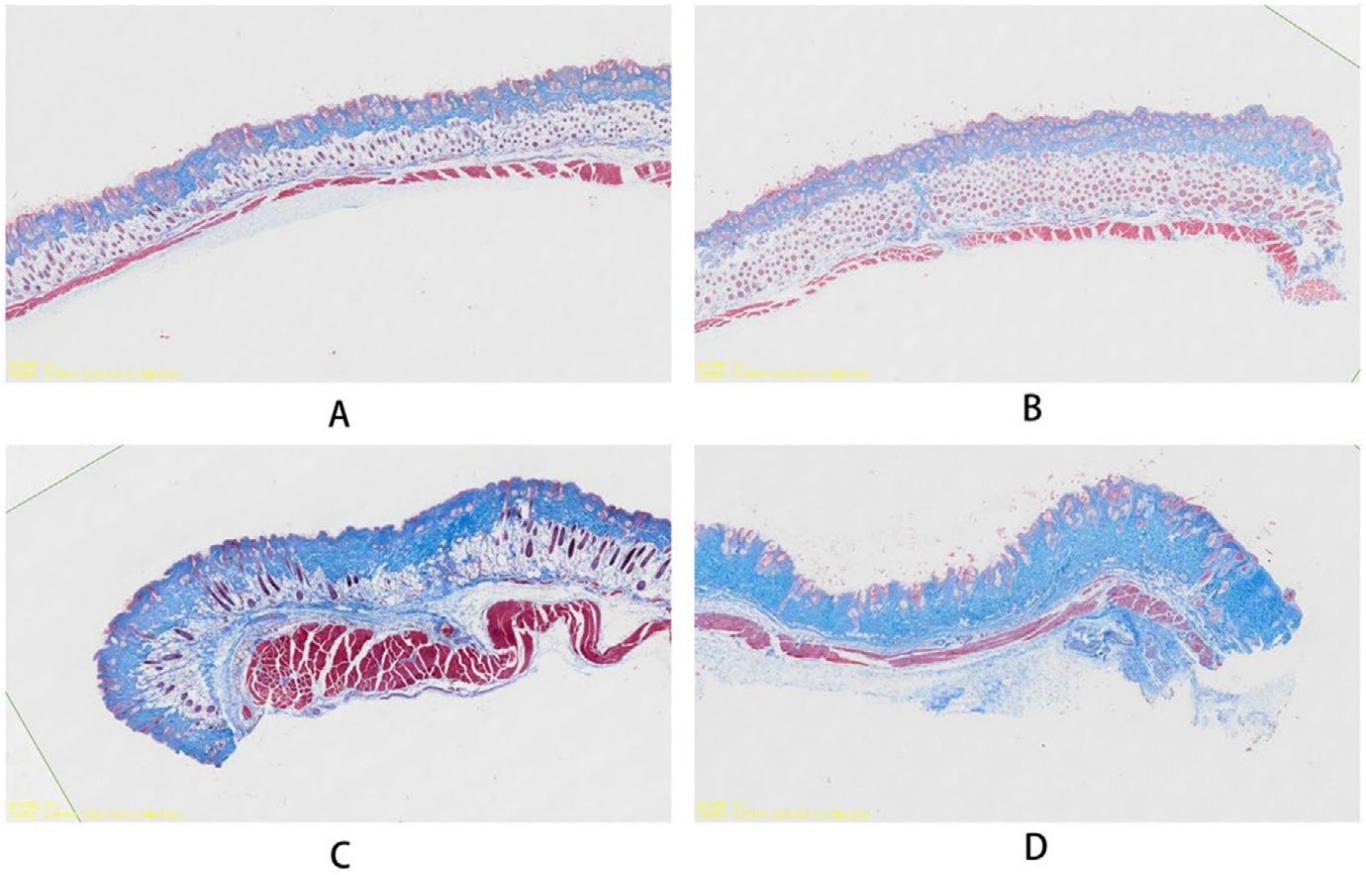
Masson's trichrome staining of mouse subcutaneous tissue sections to assess collagen content at different time points following gel injection. (A) 1 day post‐injection, (B) 1 week post‐injection, (C) 6 weeks post‐injection, (D) 8 weeks post‐injection. Blue staining indicates collagen fibers. The images demonstrate the changes in collagen deposition at the site of injection, providing insights into the remodeling process over time.

Histological analysis of the subcutaneous tissue at the injection site revealed significant changes in tissue structure and composition over time. Masson's trichrome staining was employed to specifically evaluate collagen deposition. The results demonstrated a marked increase in collagen content in the subcutaneous tissue surrounding the injection site (Figure 3A–D). At week 1, we observed the initial signs of new collagen formation, characterized by the presence of thin, loosely arranged collagen fibers. By week 6, there was a substantial increase in collagen density, with thicker and more organized collagen bundles evident. This trend continued through week 8, with the treated areas showing significantly higher collagen content compared to untreated control sites.

### 3.3 Neovascularization in the Subcutaneous Tissue

**FIGURE 4:**
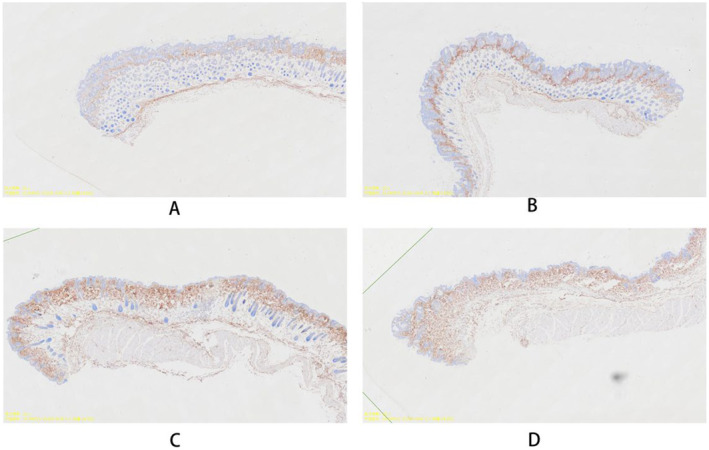
CD31 immunostaining of mouse subcutaneous tissue sections to evaluate vascular regeneration at different time points following gel injection. (A) 1 day post‐injection, (B) 1 week post‐injection, (C) 6 weeks post‐injection, (D) 8 weeks post‐injection. CD31‐positive staining indicates the presence of endothelial cells, marking blood vessel formation. These images illustrate the progression of vascular regeneration over time, indicating the dynamic changes in neovascularization post‐injection.

To assess the effect of plasma gel injection on local vascularization, we performed CD31 immunohistochemical staining to identify endothelial cells and quantify blood vessel formation (Figure 4A–D). The results showed a significant increase in vascular density in the treated areas compared to control sites.

These findings collectively demonstrate that subcutaneous injection of plasma gel not only stimulates collagen regeneration but also promotes significant neovascularization, both of which persist well beyond the period of gel absorption.

## 4 Discussion

### 4.1 Rapid Absorption of Plasma Gel

One of the most striking observations in our study was the rapid absorption of the injected plasma gel. The volume reduction of approximately 50% within the first week and near‐complete absorption by day 28 indicates that while plasma gel can provide an immediate filling effect, its persistence as a dermal filler is relatively short‐lived. This rapid absorption rate presents both challenges and opportunities for its clinical application.

The quick degradation of plasma gel can be attributed to several factors. A primary consideration is the composition of the platelet‐poor plasma (PPP) used to create the gel. A significant portion of PPP consists of water, which, although temporarily locked in place through the heating process to form a gel, is rapidly released and absorbed during the degradation process. While the gelation process provides a temporary scaffold, the high water content contributes substantially to the rapid volume loss observed in our study.

The plasma proteins, which make up a smaller fraction of the gel composition, do provide a temporary filling effect. However, these proteins are also subject to gradual absorption by the body over time. This dual process of water release and protein absorption explains the biphasic nature of the gel's degradation—an initial rapid loss of volume followed by a more gradual absorption of the remaining protein components.

Furthermore, the autologous nature of the gel means it is highly biocompatible and easily recognized by the host's immune system. This recognition likely facilitates its rapid integration and subsequent breakdown. The gel's composition, primarily consisting of plasma proteins and water, makes it susceptible to enzymatic degradation and cellular remodeling processes in the subcutaneous environment.

While the short‐term persistence of plasma gel may be seen as a limitation for long‐lasting cosmetic effects, it could be advantageous in certain clinical scenarios. For instance, in cases where temporary augmentation is desired, or in situations where a biocompatible, easily absorbed filler is preferred to synthetic alternatives. Moreover, the rapid absorption might contribute to the gel's safety profile, reducing the risk of long‐term complications associated with more persistent fillers.

To address the issue of rapid absorption, future research could focus on modifying the plasma gel composition. Strategies might include increasing the protein concentration in the PPP, incorporating cross‐linking agents to enhance the stability of the protein network, or adding components that can retain water for longer periods. These modifications could potentially extend the gel's longevity while maintaining its biocompatibility and regenerative properties.

### 4.2 Collagen Regeneration

Despite its rapid absorption, our study demonstrated a significant increase in subcutaneous collagen following plasma gel injection. This finding underscores the potential of plasma gel as a biostimulator, capable of initiating and supporting tissue regeneration processes even after its physical presence has diminished.

The observed collagen regeneration can be attributed to several mechanisms. Firstly, the plasma gel, derived from platelet‐rich plasma (PRP), contains a concentrated cocktail of growth factors and cytokines known to stimulate collagen synthesis. These include platelet‐derived growth factor (PDGF), transforming growth factor‐β (TGF‐β), and vascular endothelial growth factor (VEGF), among others. The controlled release of these factors as the gel degrades likely provides a sustained stimulus for fibroblast activation and collagen production.

Secondly, the presence of the gel itself may act as a temporary scaffold, providing a supportive environment for cellular infiltration and matrix deposition. As the gel is absorbed, it may create a favorable microenvironment for tissue remodeling, allowing for the organized deposition of new extracellular matrix components, including collagen.

The histological analysis using Masson's trichrome staining corroborated these findings, revealing increased collagen deposition in the treated areas. This increase in collagen content not only supports the gel's regenerative capabilities but also suggests potential long‐term benefits for skin quality and texture, even after the gel itself has been absorbed.

### 4.3 Neovascularization

The CD31 immunohistochemical staining results provided valuable insights into the neovascularization process following plasma gel injection. The presence of CD31‐positive cells indicates the formation of new blood vessels, a critical component of tissue regeneration and healing. This angiogenic response is likely mediated by the angiogenic factors present in the plasma gel, particularly VEGF.

Enhanced vascularization in the treated area has several important implications. Firstly, it supports the long‐term viability of the regenerated tissue by ensuring adequate blood supply and nutrient delivery. Secondly, improved vascularity may contribute to better overall skin health and appearance, potentially enhancing the cosmetic outcome beyond the initial filling effect.

The observed neovascularization also aligns with the rapid absorption of the gel, as increased blood flow to the area would facilitate the breakdown and removal of gel components. This process likely contributes to the integration of the gel with the surrounding tissue, promoting a more natural and harmonious outcome.

### 4.4 Literature Review and Comparative Analysis

The current body of research on plasma gel is relatively limited, with most studies focusing on clinical outcomes and preliminary cellular investigations. Godfrey et al. [11] conducted a multicenter retrospective study where the prepared plasma gel was injected into the deep, superficial, and dermal layers of aged facial skin in patients. The treatment regimen involved three injections spaced one month apart. Their findings indicated significant improvements in fine lines, wrinkles, and skin laxity, with all participants reporting satisfaction with the results. This study highlights the potential of plasma gel in aesthetic medicine, particularly for facial rejuvenation.

Anitua et al. [12] approached the evaluation of plasma gel from a materials science perspective, assessing its mechanical properties and rheological characteristics. They evaluated the biocompatibility, cell proliferation capacity, and effects on the migration and proliferation of human dermal fibroblasts. Their results demonstrated that the gel possesses good mechanical strength and biocompatibility, significantly promoting cell proliferation and collagen synthesis. These findings underscore the potential of PRGF technology in tissue regeneration and skin rejuvenation.

Similarly, Fedyakova et al. [7] analyzed the microstructure of the prepared gel using optical and confocal microscopy to assess its biomechanical properties. They investigated the gel's chemotactic, migratory, proliferative, and type I collagen synthesis effects on human dermal fibroblasts. Cells exposed to media containing 20% autologous gel extract showed enhanced proliferation rates and collagen production. The study confirmed that autologous protein gel exhibits promising biocompatibility and capacity to enhance cell proliferation, showing potential as a soft tissue filler and skin rejuvenation material in clinical settings.

Our study represents the first exploration of plasma gel properties using an animal model, providing a novel perspective on its application. Our findings reveal that after injection, the gel's volume‐supporting effect is transient, with half of the volume absorbed within one week and nearly all absorbed by eight weeks. This suggests that plasma gel may not be suitable as a structural filler for volume support. This rapid absorption could explain why Godfrey et al. employed a regimen of monthly injections, totaling three sessions, to maintain the clinical effect. Our clinical observations corroborate these findings, as patients often report that the filler effect diminishes significantly within one to two weeks post‐injection. Thus, while plasma gel holds promise as a filler, its limitations in sustaining volume support must be acknowledged.

Despite these limitations as a volumizing filler, plasma gel's regenerative properties are noteworthy. Both Anitua and Fedyakova, through cellular studies, demonstrated plasma gel's potential to stimulate tissue regeneration effectively, showing positive results in fibroblast proliferation and collagen synthesis. Our animal study further substantiates these findings, offering a more direct observation of plasma gel's regenerative capabilities. The evident collagen and blood vessel proliferation under the skin of nude mice eight weeks post‐injection provides compelling evidence of plasma gel's potential in promoting dermal regeneration.

### 4.5 Limitations and Future Directions

While our study provides valuable insights into the behavior of plasma gel in vivo, several limitations and areas for future research should be acknowledged. Firstly, the rapid absorption of the gel, while potentially advantageous in some scenarios, limits its efficacy as a long‐term filler. Future research should focus on developing modified plasma gel formulations with enhanced persistence. This could involve cross‐linking techniques or the incorporation of slower‐degrading components to prolong the gel's structural integrity.

Secondly, while we observed significant collagen regeneration, the long‐term stability and quality of this newly formed collagen require further investigation. Longitudinal studies extending beyond the 8‐week timeframe of our current research would provide valuable data on the durability of the regenerative effects.

Additionally, the use of nude mice as an animal model, while providing important initial data, may not fully recapitulate the human physiological response to plasma gel injection. Future studies should consider larger animal models or human clinical trials to better translate these findings to clinical practice.

Exploring the potential of combining plasma gel with other bioactive substances or cell populations could enhance its regenerative capabilities. For instance, incorporating adipose‐derived stem cells or specific growth factor cocktails might lead to more robust and sustained tissue regeneration.

Lastly, investigating the effects of repeated plasma gel injections could provide insights into the potential for cumulative benefits in collagen regeneration and tissue remodeling. This approach might offer a strategy to overcome the limitations of rapid gel absorption while maximizing the regenerative effects.

## 5 Conclusion

In conclusion, our study demonstrates that while plasma gel exhibits rapid absorption when used as a subcutaneous filler, it shows promising capabilities in promoting collagen regeneration and tissue remodeling. The observed neovascularization and collagen regeneration further support its potential as a regenerative agent.

These findings contribute significantly to our understanding of plasma gel behavior in vivo and provide a foundation for future research and development in the field of regenerative medicine and cosmetic treatments. By addressing the limitations identified in this study, particularly the rapid absorption rate, future iterations of plasma gel formulations may offer enhanced longevity while maintaining their regenerative properties, potentially revolutionizing approaches to soft tissue augmentation and regeneration.

## Author Contributions

H.C., J.L., and Y.Z. performed the research. H.C., X.X., and Y.L. designed the research study. H.C. and J.L. analyzed the data. H.C. and Y.L. wrote the paper.

## Conflicts of Interest

The authors declare no conflicts of interest.

## Data Availability

The data that support the findings of this study are available from the corresponding author upon reasonable request.
